# Quantitative Kinetic Modeling of Redox-Initiated Graft Copolymerization of MMA and Styrene onto Natural Rubber Latex

**DOI:** 10.3390/polym18091141

**Published:** 2026-05-06

**Authors:** Wanvimon Arayapranee, Weerawat Patthaveekongka

**Affiliations:** 1 Department of Chemical Engineering, College of Engineering, Rangsit University, Muang, Pathumthani 12000, Thailand; wanvimon@rsu.ac.th; 2 Department of Chemical Engineering, Faculty of Engineering and Industrial Technology, Silpakorn University, Muang, Nakhon Pathom 73000, Thailand

**Keywords:** graft copolymerization, natural rubber latex, redox initiation, power-law kinetics, multiphase reaction kinetics, interfacial polymerization

## Abstract

This study develops a quantitative kinetic framework for graft copolymerization of methyl methacrylate (MMA) and styrene (ST) onto natural rubber latex (NRL), with emphasis on Redox initiation and Interfacial polymerization in a multiphase system. Experiments were conducted using a cumene hydroperoxide/tetraethylenepentamine (CHPO/TEPA) system. Core–shell particles, consisting of a soft NR core and a rigid poly(vinyl monomer) shell, were obtained at 40–60 °C with initiator concentrations of 0.0051–0.0205 mol L^−1^ and monomer concentrations of 0.39–0.83 mol L^−1^. Radical generation occurs predominantly at the aqueous rubber interface, where monomer partitioning takes place between phases. This leads to simultaneous homopolymerization in the aqueous phase, while grafting occurs on the rubber backbone. Overall conversion (x_p_), graft conversion (x_g_), and grafting efficiency were determined gravimetrically, while morphology was confirmed by FTIR and TEM. The conversion profiles show nonlinear behavior consistent with power-law kinetics, allowing formulation of rate expressions for overall polymerization rate (R_p_) and grafting rate (R_g_). Reaction order and Arrhenius analyses indicate fractional, heterogeneous behavior characteristic of multiphase reaction kinetics. Styrene shows lower activation energy, whereas MMA exhibits higher collision frequency. The model reproduces experimental trends well (R^2^ up to 0.95) and provides insight into propagation–grafting competition in natural rubber latex systems.

## 1. Introduction

Graft copolymerization is an effective strategy for enhancing the properties of natural polymers by covalently attaching side chains that introduce functional groups, thereby improving elasticity, strength, and chemical resistance. Tanaka [1] elucidated the molecular structure of natural rubber (NR), demonstrating that its cis-1,4 configuration influences grafting behavior. Similarly, Coran and Patel [2] emphasized the importance of chemical modification and interfacial bonding in elastomer–thermoplastic systems principles that underpin modern graft copolymerization. These foundational studies underpin the chemical tailoring of NR and other natural polymers to achieve superior mechanical and interfacial properties. This versatile technique has been successfully applied to various biopolymers, including cellulose, leather, wool, and natural rubber, producing functional and sustainable materials [3,4,5]. For example, grafting butyl acrylate onto cellulose improves thermal and chemical resistance while reducing moisture absorption [5]. Chitosan grafted with PMMA via RAFT polymerization exhibits high grafting efficiency and strong adsorption of Cu(II) ions [6]. Natural rubber latex (NRL), a renewable cis-1,4-polyisoprene elastomer, contains reactive double bonds that facilitate seeded emulsion polymerization, producing core–shell particles with a soft NR core and a hard grafted shell. The NR core imparts flexibility, whereas the grafted shell enhances strength, oil resistance, and interfacial compatibility [7,8]. Mohapatra & Nando [9,10] demonstrated that CHPO/TEPA redox initiation enables grafting reactions in natural rubber latex under mild conditions, highlighting the suitability of redox systems for controlled graft copolymerization in dispersed rubber environments. A wide variety of vinyl monomers including methyl methacrylate (MMA), styrene (ST), acrylonitrile (AN), glycidyl methacrylate (GMA), and maleic anhydride (MA) have been grafted onto NR latex to improve compatibility and performance. MMA yields stable NR-g-PMMA latexes for adhesive and impact-resistant products [11]. Earlier kinetic investigations by Ghosh and Sengupta [12] demonstrated that both monomer and initiator concentrations significantly influence the grafting rate and efficiency in MMA-grafted natural rubber systems, highlighting the coupled roles of radical generation and monomer availability. These findings underscore the importance of quantitative kinetic modeling in understanding MMA-based graft copolymerization. ST and AN grafting enhance modulus, barrier, and thermal resistance [13,14,15], while compatibilizing monomers such as GMA and MA promote interfacial adhesion in NR/PMMA and PLA/NR blends [16,17]. The kinetics of these grafting systems have been investigated extensively to elucidate radical mechanisms and rate-determining steps. Taghizadeh and Ghaffari [18] reported that for MMA grafting onto dehydrochlorinated PVC using benzoyl peroxide (BPO), the rate (R_g_) was found to be k [MMA]^0.977^ [DHPVC]^0.545^ [BPO]^0.545^, with an activation energy of 37.44 kJ·mol^−1^ in the temperature range of 40–70 °C. Similarly, Taghizadeh and Mehrdad [19] found that acrylic acid and ethyl methacrylate grafting onto starch using ceric ammonium nitrate (CAN) followed R_g_ = k [AA]^0.92^ [CAN]^0.56^ [St]^0.46^ and R’_g_ = k’ [EMA]^0.92^ [CAN]^0.53^ [St]^0.48^, with activation energies of 36.25 kJ·mol^−1^ (20–40 °C) and 11.88 kJ·mol^−1^ (25–45 °C), respectively. In NRL systems, grafting efficiency of MMA and ST is strongly influenced by initiator concentration and monomer feed ratio, which determine radical formation, graft yield, and particle morphology [20,21]. For hydrophilic polysaccharide systems, grafting behavior is controlled by both initiator decomposition and monomer diffusion, reflecting the coexistence of diffusion- and reaction-controlled regimes [22,23]. Such kinetic insights emphasize the importance of developing reliable rate laws to optimize grafting performance. Polystyrene-grafted NR (PS/NR) composites further demonstrate improved compatibility between immiscible polymers, with the NR core serving as a toughening phase and the PS shell enhancing interfacial adhesion [24]. By adjusting initiator and monomer concentrations and polymerization temperature, graft copolymers can be tailored to exhibit specific particle sizes, shell thicknesses, grafting efficiencies, and chemical compositions. These parameters significantly affect reaction kinetics and morphology. Importantly, the process of grafting MMA onto NR has been scaled up from laboratory to pilot plant production in Southern Thailand using a CHPO/TEPA redox system [11]. Despite this progress, detailed kinetic studies on NR grafting remain limited. Rate models correlating initial polymerization rate with grafting parameters have been proposed under steady-state conditions typically within 10 to 240 min of reaction depending on the monomer backbone combination [20,21]. Accordingly, this work focuses on investigating the kinetics of graft copolymerization in the formation of natural rubber (NR) core/poly(vinyl monomer) shell composites initiated by the CHPO/TEPA redox system. The effects of reaction time, temperature, cumene hydroperoxide concentration, monomer-to-rubber ratio, and monomer type on the overall copolymerization rate, grafting rate, and grafting efficiency were systematically examined. Furthermore, rate equations describing both the overall copolymerization and grafting reactions were established as power-law functions of these process variables. The reaction orders and Arrhenius parameters were determined by fitting the proposed rate equations to experimental data obtained under various reaction conditions, using natural logarithmic transformations and linear regression. In this study, styrene (ST) and methyl methacrylate (MMA) were selected as representative monomers due to their widespread use in NR grafting.

## 2. Materials and Methods

### 2.1. Materials

The NR latex used in this work was commercial high-ammonia grade, having 60% dry rubber content (DRC), provided by Thai Hua Rubber Public Company Ltd., Rayong, Thailand. MMA and ST monomers (Reagent grade ≥ 99%; Aldrich, St. Louis, MO, USA) were purified by washing with a 10% aqueous solution of sodium hydroxide (~99%; Caledon, Georgetown, ON, Canada) followed by deionized water until neutral. Sodium lauryl sulfate (SDS, ≥99%; Aldrich, St. Louis, MO, USA) was used as received as a surfactant, isopropanol (99.5%; EMD, Oakville, ON, Canada) as a stabilizer, potassium hydroxide (KOH, ~85%; BDH Inc., Toronto, ON, Canada) as a buffer, and CHPO (~80%; Aldrich, St. Louis, MO, USA) as an initiator for the redox initiator system containing TEPA (~85%; Aldrich, St. Louis, MO, USA) as an activating agent. Deionized (DI) water was used for all solution preparations.

### 2.2. Preparation of Grafted NR

The reaction was carried out using a batch emulsion graft copolymerization technique. NR latex (40 g) was used as the rubber substrate in all experiments and was kept constant throughout the study. The polymerization recipes were designed to investigate the kinetic behavior of the graft copolymerization over the temperature range of 40–60 °C. The reaction time was varied up to 480 min. The initial monomer concentration was varied over the range 0.39–0.83 M and is denoted [M]_0_. The rubber concentration, [*R*], was defined as the concentration of carbon–carbon double bonds in the NR structure, which was calculated based on the effective rubber content in the latex, the total reaction volume, and the molecular weight of the isoprene repeating unit (68.02 g mol^−1^), as described below:

(1) $$\left[R\right],\;\left(\mathrm{mol}/L\right)=\;\frac{\mathrm{wt}.\;\mathrm{dry}\;\mathrm{rubber}\;(g)}{\mathrm{reaction}\;\mathrm{volume}\;(L)\times 68.02}$$

Since the CHPO/TEPA system consists of a hydrophobic oxidizing agent and a hydrophilic reducing agent, their interaction generates both TEPA radicals and cumyloxy radicals, with the latter being the primary species responsible for initiation [25]. Accordingly, the CHPO-to-TEPA molar ratio was fixed at unity throughout the study. The CHPO concentration varied in the range of 0.0051–0.0205 M. It should be noted that the solid content of the reaction mixture was maintained at 20 wt% under all experimental conditions. The polymerization recipes and reaction conditions are summarized in Table 1.

The stock emulsion was first prepared in a 500 mL glass reactor equipped with a mechanical stirrer. SDS (1.5 phr, parts per hundred parts of dry rubber) was dissolved in the required amount of deionized (DI) water and charged into the reactor while stirring at 400 rpm. Subsequently, 5 phr of isopropanol and the desired amount of NR latex were added sequentially. The resulting mixture was deoxygenated by purging with nitrogen gas for approximately 30 min. To maintain the latex pH above 10, 0.5 phr of KOH was added after the mixture had been stirred for 15 min. The desired amount of monomer and the TEPA activator were then introduced into the reactor, and the NR seed particles were allowed to swell for 1 h. Thereafter, 30 mL of the stock emulsion was transferred into 100 mL glass reaction bottles, which were sealed with rubber septa. The reaction bottles were prepared for two experimental series based on the sampling times. In Series 1, bottles were withdrawn at 60, 180, 300, and 420 min, whereas in Series 2, sampling was carried out at 30, 120, 240, 360, and 480 min. Each bottle was placed in a water bath that was shaken at the designated reaction temperature. Subsequently, CHPO was injected into each bottle, and the reaction time was recorded. At the predetermined time, the bottle was withdrawn and immediately immersed in an ice-cold water bath to quench the reaction. The resulting latex was coagulated using boiling water containing 5 wt% formic acid, followed by repeated washing with DI water. The coagulated product was then dried in a vacuum oven at 50 °C until a constant weight was achieved. The weights of the latex samples before and after drying were recorded and used to calculate the fractional overall polymer conversion (*x_p_*), which includes both graft copolymer and free homopolymer, according to Equation (2).

(2) $$\mathrm{Fractional}\;\mathrm{overall}\;\mathrm{polymer}\;\mathrm{conversion}\;\left(x_{p}\right)=\;\frac{\mathrm{wt}.\;\mathrm{of}\;\mathrm{monomer}\;\mathrm{polymerized}}{\mathrm{wt}.\;\mathrm{of}\;\mathrm{initial}\;\mathrm{monomer}\;}$$

The free homopolymer in the product sample was removed by Soxhlet extraction. The sample was extracted with a 50:50 (%v) mixture of acetone/methyl ethyl ketone for 24 h and dried in a vacuum oven at 50 °C for another 24 h. The residual product is called graft copolymer or grafted NR. The fractional conversions of the graft copolymer and free homopolymer, as well as grafting efficiency, were determined gravimetrically according to the relationship:

(3) $$\mathrm{Fractional}\;\mathrm{graft}\;\mathrm{copolymer}\;\mathrm{conversion}\;(x_{g})\;=\;x_{p\;}-\;\frac{\mathrm{wt}.\;\mathrm{of}\;\mathrm{extracted}\;\mathrm{polymer}\;\mathrm{by}\;\mathrm{soxhlet}}{\mathrm{wt}.\;\mathrm{of}\;\mathrm{initial}\;\mathrm{monomer}\;}$$

(4) $$\mathrm{Fractional}\;\mathrm{free}\;\mathrm{homopolymer}\;\mathrm{conversion}\;(x_{f})\;=\;x_{p}\;-\;x_{g}$$

(5) $$\mathrm{Grafting}\;\mathrm{efficiency}\;(\mathrm{GE},\%)=\frac{x_{g}}{x_{p}\;}\text{ × }100$$

The overall copolymerization rate and the grafting rate at any time during the reaction were calculated from the slopes of the *x_p_*^−t^ and *x_g_*^−t^ curves at that time, respectively. After Soxhlet extraction, the formation of graft copolymer was characterized using a Perkin Elmer Frontier Fourier Transform Infrared (FTIR) spectrometer, based on a Universal Attenuated Total Reflectance (UATR) FTIR with ZnSe-Diamond composite crystal. The spectra were recorded at room temperature with a resolution of 4 cm^−1^ and 4 scans, in the spectral range of 4000 to 650 cm^−1^.

The morphologies of the neat and grafted rubber were observed. The latex sample was diluted 400 times with DI water. A drop of the diluted solution was applied to a 400-mesh copper grid and then exposed to vapor from 1 mL of 2% osmium tetroxide solution overnight. The stained grids were examined by using a CM 10 PW6020/10 Transmission Electron Microscope (Philips, Eindhoven, The Netherlands) at 100 kV.

## 3. Results and Discussions

### 3.1. Characterization of Grafted NR

**Figure 1:**
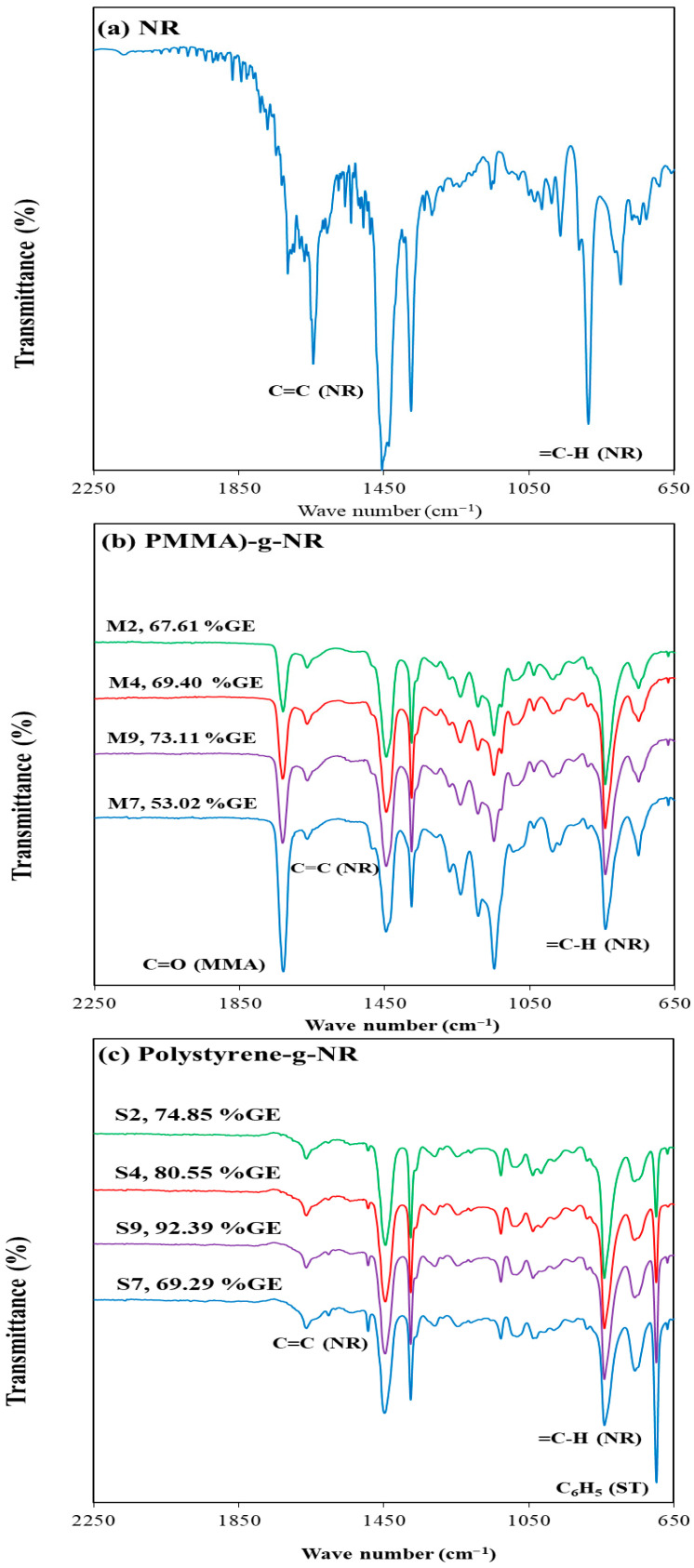
FTIR spectra of (**a**) NR, (**b**) NR-g-PMMA (8 h), and (**c**) NR-g-polystyrene (8 h).

FTIR analysis was employed to identify the functional groups of grafted NR after Soxhlet extraction and to compare them with neat NR. As shown in Figure 1a, neat NR exhibits characteristic bands at 840 and 1664 cm^−1^, corresponding to the =CH out-of-plane bending and C=C stretching vibrations of cis-1,4-polyisoprene, respectively. In contrast, the spectra of PMMA and PST-grafted NR (Figure 1b,c) display new absorption bands at 1732 and 698 cm^−1^, assigned to the C=O stretching of ester groups and the monosubstituted benzene ring, respectively, confirming successful grafting of vinyl monomers onto the NR chains [26,27]. The intensities of these characteristic bands generally increase with increasing grafting efficiency (%GE). However, samples prepared at a higher monomer concentration (0.83 M; M7 and S7) show higher absorption intensities despite lower %GE compared with those prepared at 0.39 M. This behavior reflects the increased monomer concentration, which enhances monomer incorporation onto the NR chains while simultaneously promoting free homopolymer formation, thereby reducing %GE at high monomer loadings. As the monomer concentration increases, the formation of free homopolymer becomes increasingly favored over graft copolymerization (as discussed in the following section), leading to a corresponding decrease in grafting efficiency (GE).

**Figure 2:**
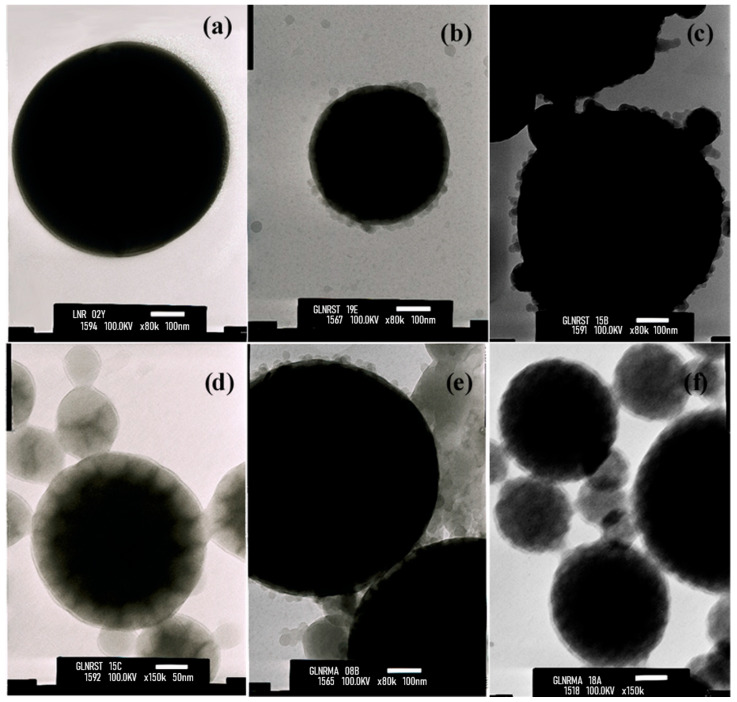
TEM images of (**a**) NR; grafted NR with polystyrene at different grafting efficiencies (GE = 33.02% (**b**), 42.86% (**c**), and 80.55% (**d**)); and grafted NR with PMMA at GE = 39.70% (**e**) and 69.40% (**f**).

The particle morphology was examined by TEM (Figure 2). Rubber particles can be classified by size into small (10–250 nm) and large (250–3000 nm) particles [28,29]. The pristine NR particles [Figure 2a] exhibit a smooth, uniform surface. After grafting, distinct core–shell structures become evident for both polystyrene-grafted NR [Figure 2b–d] and PMMA-grafted NR [Figure 2e,f] at different grafting efficiencies (GE, %). In these images, the darker regions correspond to the NR core, while the lighter contrast indicates the grafted polymer shell. At low GE, small nodular domains are observed on the particle surface, indicating the early stage of graft formation. As GE increases, these domains grow and gradually merge, forming a more continuous shell around the NR core. In the PMMA system, this effect is more pronounced, with higher GE resulting in more complete encapsulation. The shell thickness is generally below 50 nm and increases with increasing GE, consistent with progressive graft growth.

### 3.2. Rate Equations

After adding CHPO/TEPA into the reaction, CHPO is reduced by TEPA to generate radicals (RO^•^), including cumyloxyl, hydroxyl, and dimethyl phenyl carbinol radicals [10,30]. In redox-initiated emulsion systems, radical generation is commonly associated with interfacial regions where oxidizing and reducing components interact [25], consistent with classical descriptions of redox initiation in radical polymerization [31]. The resulting alkoxy radicals can initiate NR chains, producing polyisoprene radicals (Equation (7)), which subsequently react with monomer to form graft macroradicals during polymerization (Equation (9)). Simultaneously, these radicals may initiate monomer molecules, generating free homopolymeric radicals (Equations (6) and (8)), which can further interact with polyisoprene radicals to yield a graft copolymer (Equation (15)). The homopolymeric radicals and graft macroradicals can transfer to monomer and to rubber (Equations (10)–(13)), yielding homopolymer and graft copolymer [30]. Moreover, some of the free homopolymeric radicals can terminate by combination to produce a free homopolymer (Equation (14)).

**Figure 3:**
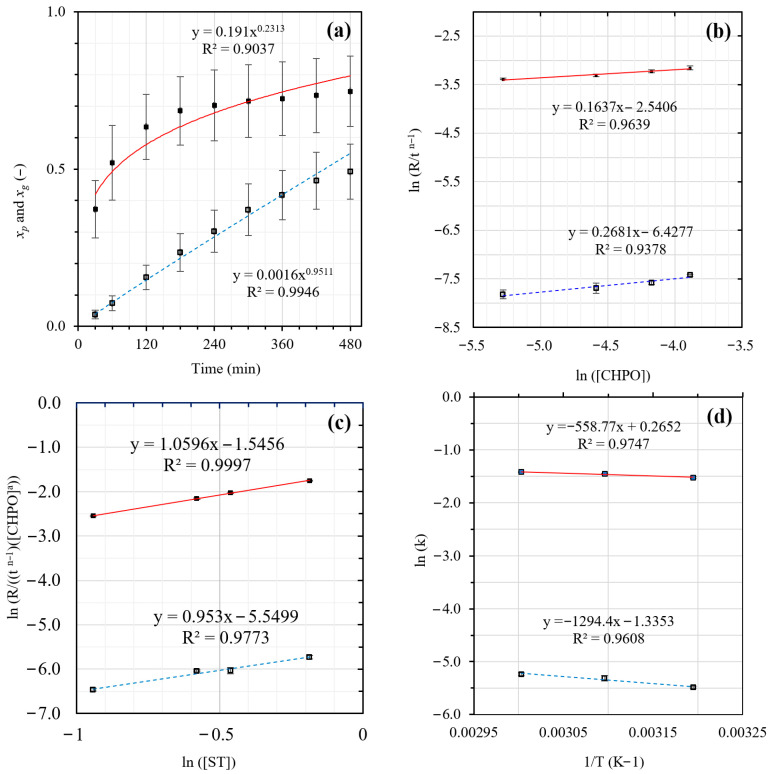
Kinetic analysis of MMA grafting onto NR: (**a**) time evolution of *x_p_* (■) and *x_g_* (□); (**b**) effect of [CHPO]_0_; (**c**) effect of [MMA]_0_; and (**d**) effect of reaction temperature.

Based on the observed conversion-time curves (Figure 3) for the overall polymer (*x_p_*), graft copolymer (*x_g_*), and free homopolymer (*x_f_*). During the early stages of the reaction (0–150 min), the *x_f_* curve shows a trend similar to that of *x_p_*; conversion increases rapidly with time. Since *x_p_* is the summation of *x_g_* and *x_f_*, it indicates that most of the monomer polymerizes to produce free homopolymeric radicals during the early stages of the reaction. After 150 min, when *x_p_* starts to level off, *x_g_* continuously increases with time, but *x_f_* decreases. This phenomenon implies that the free homopolymeric radicals produced readily bind to the NR backbone, thereby forming a graft copolymer.

The reaction mechanisms of initiator decomposition, chain initiation, chain propagation, and graft copolymerization termination for NR/MMA or ST can be summarized as follows.

Chain initiation:

(6) $$RO^{•}\;+\;M\to M_{1}^{•}$$

(7) $$RO^{•}\;+\mathrm{NR}\to {NR}^{*}$$

Chain propagation:

(8) $$M_{1}^{•}\;+\;M\to M_{2}^{•}\;\vdots \;\vdots \;\;M_{n-1}^{•}\;+\;\;M\to M_{n}^{•}\;\;{NR}^{•}+M\to \mathrm{NR}M_{1}^{•}\;\vdots \;\vdots$$

(9) $$\mathrm{NR}M_{n-1}^{•}+M\to \mathrm{NR}M_{n}^{•}$$

Chain transfer:

(10) $$M_{n}^{•}+M\to M_{n}+M^{•}$$

(11) $$\mathrm{NR}M_{n}^{•}+M\to \mathrm{NR}M_{n}+M^{•}$$

(12) $$M_{n}^{•}+\mathrm{NR}\to M_{n}+NR^{•}$$

(13) $$\mathrm{NR}M_{n}^{•}+\mathrm{NR}\to \mathrm{NR}M_{n}+NR^{•}$$

Chain termination:

(14) $$M_{n}^{•}+M_{m}^{•}\to M_{n+m}(\mathrm{Homopolymer})$$

(15) $$\mathrm{NR}M_{n}^{•}+M_{m}^{•}\to \mathrm{NR}M_{n+m}(\mathrm{Graft}\;\mathrm{copolymer})$$

where M is either MMA or ST monomer; NR is polyisoprene chain (NR chain); and M^•^ and NRM^•^ are free homopolymeric radical and graft macroradical, respectively.

**Figure 4:**
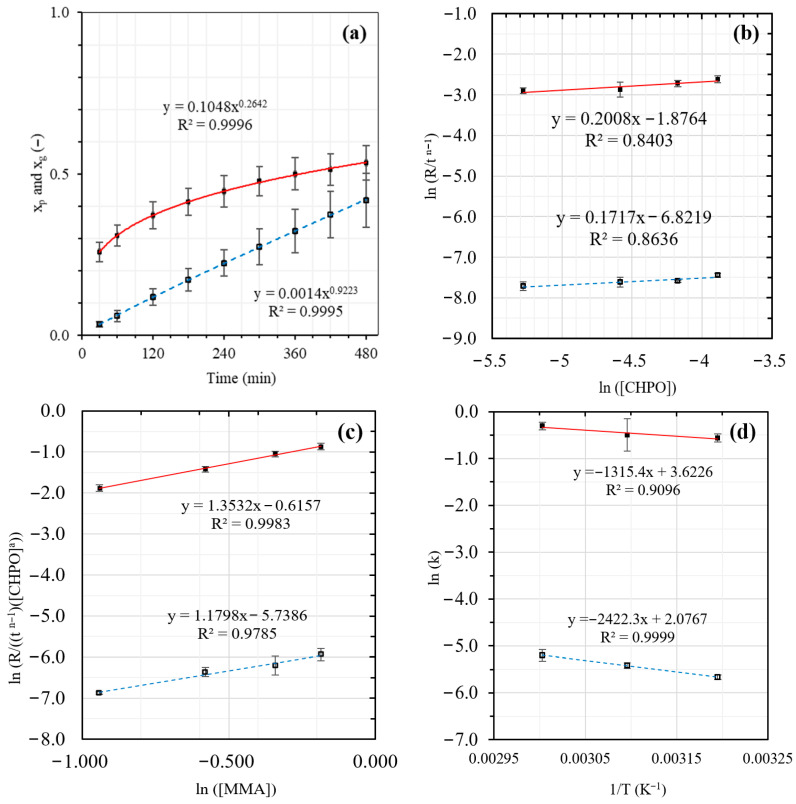
Kinetic analysis of ST grafting onto NR: (**a**) time evolution of *x_p_* (■) and *x_g_* (□); (**b**) effect of [CHPO]_0_; (**c**) effect of [ST]_0_; and (**d**) effect of reaction temperature.

According to the conversion-time profiles, *x_p_* and *x_g_* are a nonlinear function of time (t). Both can be well fitted to a power function, as shown in Figure 3a and Figure 4a. Therefore, the relation of conversion and time can be written as

(16) $$x=Kt^{n}$$

Differentiating with t and multiplying by M_0_, gives

(17) $$M_{0}\frac{dx}{dt}=kt^{n-1}$$

where k = M_0_Kn, and K and n are empirical constants determined from the conversion time relationship. M_0_ is the initial monomer concentration (mol L^−1^). Based on the relationship of $R=-d\left[M\right]/dt$, the rate equation can be defined by

(18) $$R_{p}=k_{p}t^{n_{p}-1};\;\mathrm{for}\;\mathrm{overall}\;\mathrm{copolymerization}\;\mathrm{rate}$$

(19) $$R_{g}=k_{g}t^{n_{g}-1};\;\mathrm{for}\;\mathrm{grafting}\;\mathrm{rate}$$

where the subscripts p and g represent overall copolymerization and grafting reactions, respectively. As previously discussed, process factors initiator concentration, monomer concentration, rubber concentration, and temperature significantly influence graft copolymerization. These variables have been proposed to relate to the rate through a power-law dependence [19,22]. Therefore, Equations (18) and (19) can be rewritten as:

(20) $$R_{p}=k_{p}[CHPO{]}^{a_{p}}[M{]}^{b_{p}}t^{n_{p}-1}$$

(21) $$R_{g}=k_{g}[CHPO{]}^{a_{g}}[M{]}^{b_{g}}t^{n_{g}-1}$$

where [CHPO] and [M] represent the initial initiator (mol L^−1^) and monomer (MMA or ST) concentrations (mol L^−1^), respectively. The superscripts a, and b denote the corresponding reaction orders. According to the Arrhenius relation, k = A exp(−E/RT), where A, E, R, and T are the pre-exponential factor, activation energy, gas constant, and temperature, respectively. Substituting this relation into Equations (20) and (21) gives:

(22) $$R_{p}=A_{p}e^{-E_{p}/RT}[CHPO{]}^{a_{p}}[M{]}^{b_{p}}t^{n_{p}-1}$$

(23) $$R_{g}=A_{g}e^{-E_{g}/RT}[CHPO{]}^{a_{g}}[M{]}^{b_{g}}t^{n_{g}-1}$$

Generally, the rate equation expressed as a power function of the process variables is independent of time. The reaction orders and Arrhenius parameters were determined from natural logarithmic plots using linear regression, since the proposed rate expression exhibits a nonlinear dependence on time. The nonlinear conversion behavior observed in this study agrees well with the findings of Mohapatra and Nando [10], who demonstrated that CHPO/TEPA-initiated grafting reactions follow a power-law dependence on reaction variables such as time and initiator concentration. Prior to conducting these analyses, Equations (22) and (23) were rearranged as follows:

(24) $$\frac{R_{p}}{t^{n_{p}-1}}=A_{p}e^{-E_{p}/RT}[CHPO{]}^{a_{p}}[M{]}^{b_{p}}$$

(25) $$\frac{R_{g}}{t^{n_{p}-1}}=A_{g}e^{-E_{g}/RT}[CHPO{]}^{a_{g}}[M{]}^{b_{g}}$$

Therefore, the rate per t^n−1^ shown in Equations (24) and (25) remains constant at any reaction time.

### 3.3. Effect of Reaction Time

Figure 3 and Figure 4, corresponding to the MMA and ST systems, respectively, summarize the kinetic analyses used to derive the rate expressions for overall polymerization (R_p_) and grafting (R_g_) encompassing (a) effect of reaction time, (b) effect of initiator concentration, (c) effect of monomer concentration, and (d) effect of reaction temperature (Arrhenius plots). Based on Figure 3a and Figure 4a, the evolutions of the overall polymer conversion (*x_p_*) and graft copolymer conversion (*x_g_*) are presented with error bars reflecting the combined effects of variations in CHPO concentration (0.0051–0.0205 M), monomer concentration (0.39–0.83 M), and reaction temperature (313–333 K) at fixed reaction times (30–480 min). In contrast, for Figure 3b–d and Figure 4b–d, error bars were derived from conversion values pooled over reaction times from 30 to 480 min, thereby reflecting the overall variability of the system across the investigated process conditions, including contributions from both time evolution and process parameter variations.

Power law fitting of the conversion time data reveals nonlinear time dependences for both processes. The overall polymer conversion increases rapidly at early reaction times (t < 180 min) due to high monomer availability and efficient radical generation, but gradually slows and approaches a quasi-plateau at longer times as monomer depletion, increased viscosity, and termination effects become significant. In contrast, *x_g_* increases steadily throughout the reaction without a pronounced initial acceleration, consistent with grafting being a secondary process governed by the generation of active macroradicals on the rubber backbone. Both *x_p_* and *x_g_* exhibit nonlinear time dependences that are well described by power law relationships (Figure 3a and Figure 4a). For the MMA system, the fitted time exponents are n_p_ = 0.2313 and n_g_ = 0.9511, while for the ST system, n_p_ = 0.2642 and n_g_ = 0.9223. The CHPO/TEPA system functions as a redox initiator predominantly distributed in the aqueous phase, where efficient radical generation occurs via redox decomposition. Methyl methacrylate (MMA), owing to its polar ester group (-COOCH_3_), exhibits appreciable solubility and mobility in the aqueous phase in addition to its partitioning into the rubber phase. This preferential availability of MMA in the aqueous phase promotes rapid early-stage overall polymerization, including homopolymerization, leading to a higher overall polymer conversion (*x_p_*) than graft copolymer conversion (*x_g_*). Therefore, overall polymerization in the MMA system reaches diffusion- and termination-limited regimes at earlier stages, resulting in a lower apparent time exponent (n_p_) compared with styrene (ST). In contrast, MMA can more readily supply monomer to the aqueous–rubber interfacial region, where NR macroradicals are generated, allowing grafting to proceed cumulatively over time and yielding a slightly higher ng than observed for ST. For the ST system, grafting becomes increasingly diffusion- and locus-limited within the rubber phase as the grafted shell develops, thereby reducing the apparent time dependence of graft copolymer formation.

### 3.4. Effect of Initiator Concentration

Figure 3b and Figure 4b show the ln–ln relationships between $R_{p}/t^{\left(n_{p}-1\right)}$, $R_{g}/t^{n_{g}-1}$, and the initial CHPO concentration for MMA and styrene graft copolymerization onto NR, from which the apparent reaction orders were determined. The different sensitivities of $R_{p}/t^{\left(n_{p}-1\right)}$ and $R_{g}/t^{n_{g}-1}$ to CHPO concentration can be attributed to the interfacial nature of the CHPO/TEPA redox initiation system, as well as to monomer phase partitioning in the emulsion medium. In this system, CHPO preferentially associates with the rubber phase, whereas TEPA resides mainly in the aqueous phase; consequently, radical generation occurs predominantly at the aqueous–rubber interface, where radicals may either initiate polymerization within the polymerization loci (e.g., micelles or growing particles) or generate active sites on the NR backbone that subsequently initiate graft chains. The reaction orders with respect to CHPO were 0.2008 (a_p_) for R_p_/t^−0.7687^ and 0.1717 (a_g_) for R_g_/t^−0.0489^ in the MMA system, whereas the corresponding values in the styrene system were 0.1637 (a_p_) and 0.2681 (a_g_) for R_p_/t^−0.7358^ and for R_g_/t^−0.0777^, respectively. These results indicate that increasing initiator concentration promotes overall polymerization slightly more strongly than graft propagation in the MMA system, whereas in the styrene system, grafting becomes more sensitive to initiator concentration. For MMA, increasing CHPO mainly enhances the overall radical flux, favoring chain initiation and propagation within the polymerization loci and thus promoting conventional emulsion polymerization relative to graft growth on the NR backbone. In contrast, the stronger CHPO dependence of R_g_ observed for styrene suggests that higher initiator concentrations facilitate the formation of radical sites on or near the NR particle surface, possibly through enhanced allylic activation of the NR backbone, thereby increasing the probability of graft initiation and growth. This behavior is also consistent with the physicochemical properties of the monomers: styrene, being more hydrophobic, preferentially partitions into the rubber particles and increases the likelihood of radical interaction with the NR backbone, whereas MMA shows greater affinity for the aqueous phase and polymerization loci, favoring bulk polymerization pathways. The relatively weaker CHPO dependence of R_g_ in the MMA system further suggests that a fraction of the generated radicals participate in competing pathways, such as homopolymer initiation or radical termination, rather than contributing to effective graft propagation. Consequently, although increasing CHPO enhances radical generation, excessive initiator concentrations may also promote radical–radical recombination and the formation of free copolymers, thereby limiting the effective participation of radicals in graft propagation. Similar behavior has been reported in graft copolymerization systems, where excessive initiator concentrations increase free homopolymer formation and decrease grafting efficiency due to preferential termination of free homopolymer radicals over grafting onto the rubber backbone [32]. More generally, the influence of initiator concentration in grafting systems is often evaluated through grafting efficiency (GE), which represents the fraction of radicals contributing to graft copolymer formation, and many studies report an optimal initiator concentration beyond which GE decreases because of enhanced free polymerization and radical termination [33,34,35]. Although these studies focus on GE rather than grafting rate, a decrease in GE mechanistically reflects reduced radical participation in graft propagation. The present kinetic results are consistent with this interpretation, indicating that increasing CHPO increases radical generation but does not proportionally increase R_g_ because of competing radical pathways and interfacial constraints; however, the present system remains within the radical-increasing regime, and the literature-reported decrease in GE is cited here as mechanistic support rather than a direct experimental observation.

### 3.5. Effect of Monomer Concentration

ln–ln analyses with respect to the initial monomer concentration (Figure 3c and Figure 4c) yield apparent reaction orders of 1.3532 (b_p_) for R_p_/(t^−0.7687^ [CHPO]^00.2008^) and 1.1798 (b_g_) for R_g_/(t^−0.0489^ [CHPO]^00.1717^) in the MMA system, indicating strong monomer dependence for both overall polymerization and grafting. The higher sensitivity of R_p_ relative to R_g_ suggests that increasing monomer concentration mainly enhances chain propagation within the polymerization loci, whereas graft formation remains partially constrained by interfacial factors at the NR particle surface, such as the availability of active radical sites and steric limitations from the growing graft layer. In comparison, styrene exhibits lower monomer reaction orders for both R_p_/(t^−0.7358^ [CHPO]^00.1637^) = 1.0596 (b_p_) and R_g_/(t^−0.0777^ [CHPO]^00.2681^) = 0.953 (b_g_) than MMA, indicating that polymerization and grafting in the styrene system are less sensitive to changes in bulk monomer concentration. This difference can be attributed to monomer partitioning behavior in the emulsion system. Styrene, being highly hydrophobic, preferentially partitions into the rubber particles, leading to relatively high local monomer concentrations near the NR phase even at moderate bulk monomer levels. Consequently, further increases in bulk styrene concentration produce only a limited additional effect on propagation and grafting kinetics. In contrast, MMA exhibits higher aqueous solubility due to its polar ester group (-COOCH_3_), enabling efficient diffusion through the aqueous phase toward the polymerization loci; therefore, variations in MMA concentration more directly influence monomer availability for propagation and graft growth. Although increasing monomer concentration increases the probability of propagation and graft-site formation, excessive monomer levels may also promote competing reactions, such as chain transfer and free polymerization [36,37]. In grafting systems, these competing pathways are often reflected by a decrease in grafting efficiency (GE). Moreover, surface-controlled mechanisms where polymerization occurs primarily at NR particle surfaces and the growing grafted shell gradually restricts monomer diffusion [38,39] may further limit effective graft propagation at elevated monomer concentrations. Within the investigated monomer range, however, both R_p_ and R_g_ increase monotonically, as indicated by the positive reaction orders in Figure 3c and Figure 4c. Therefore, the literature-reported decline in GE is invoked here as mechanistic support for radical competition and diffusion constraints rather than as a direct experimental observation. Collectively, these results indicate that although monomer concentration influences propagation kinetics, effective graft formation is ultimately governed by monomer partitioning and interfacial accessibility at the NR particle surface, consistent with the findings of Wang et al. [40], who reported higher grafting efficiency in systems exhibiting improved monomer–polymer compatibility.

### 3.6. Effect of Reaction Temperature

The temperature dependences of R_p_ and R_g_ follow Arrhenius behavior, as shown in Figure 3d and Figure 4d. The apparent activation energies were determined from the slopes of the natural logarithm plots (ln k vs. 1/T, slope = –E/R), and the pre-exponential factors were obtained from the corresponding intercepts. As summarized in Table 2, styrene exhibits lower apparent activation energies than MMA for both propagation (E_p_ = 4.64 vs. 10.94 kJ mol^−1^) and grafting (E_g_ = 10.76 vs. 20.14 kJ mol^−1^), indicating energetically more favorable elementary reaction steps. The relatively low apparent activation energies obtained in this study are consistent with redox initiated radical polymerization systems, in which chemical initiation pathways enable polymerization at moderate temperatures compared with purely thermal initiation [31,41,42]. Such low apparent activation energies are characteristic of redox-initiated systems, because chemical radical generation lowers the effective energy barrier for initiation and reduces the overall temperature dependence of the reaction rate. In redox emulsion systems, radical generation is often associated with interfacial regions where oxidizing and reducing species interact [25], and increasing temperature may further enhance molecular mobility and monomer diffusion, thereby influencing interfacial radical encounters and effective propagation rates. A comparable activation energy was reported by Singha et al. [43], who obtained an E_g_ value of 12.48 kJ mol^−1^ for MMA grafting onto cellulose fibers using an ascorbic acid/H_2_O_2_ redox system, further supporting the notion that low activation barriers are typical for redox-mediated grafting processes.

However, activation energy alone does not fully account for the observed kinetics. Despite its lower E_p_, styrene exhibits a substantially smaller pre-exponential factor for propagation (A_p_ = 1.30 vs. 37.43 for MMA), suggesting that radical–monomer encounters are frequency-limited within the rubber phase. In contrast, the larger A_p_ value for MMA reflects more frequent effective collisions, consistent with greater monomer mobility in the aqueous phase. A similar contrast is observed for grafting: although styrene possesses a lower E_g_, its pre-exponential factor (A_g_ = 0.26) is markedly smaller than that of MMA (A_g_ = 7.98), indicating that graft formation in the styrene system is primarily constrained by radical backbone encounter frequency and interfacial accessibility rather than apparent activation barriers. Collectively, these results support the coupled energetic and transport-controlled nature of graft copolymerization in multiphase latex systems, where activation barriers and radical encounter frequency jointly determine the effective reaction rate.

In the present kinetic formulation, the initiator concentration was explicitly coupled with the temperature dependent decomposition of the CHPO/TEPA redox system rather than treated as a fixed parameter. The effective initiator concentration was defined as:

(26) $${\left[CHPO\right]}_{eff}=k_{d}\left(T\right){\left[CHPO\right]}_{0}$$

For MMA polymerization:

(27) $${\left[CHPO\right]}_{eff}=(3.48\times 10^{6})exp(-\frac{52,990}{RT}){\left[CHPO\right]}_{0}$$

For styrene polymerization:

(28) $${\left[CHPO\right]}_{eff}=(6.70\times 10^{11})exp(-\frac{86,180}{RT}){\left[CHPO\right]}_{0}$$

where the Arrhenius parameters were adopted from previously reported kinetic analysis [44]. Incorporation of these system-specific decomposition constants enables explicit representation of temperature sensitive radical generation within the multiphase latex environment.

Accordingly, increasing temperature accelerates initiator decomposition and enhances cumyloxy radical formation [31,45], thereby increasing radical flux and elevating both R_p_ and R_g_, consistent with the positive Arrhenius trends observed in Figure 3d and Figure 4d. Elevated temperature further promotes monomer diffusion by enhancing molecular mobility and reducing the medium’s viscosity [46,47,48,49], thereby strengthening interfacial radical–monomer interactions. Temperature therefore governs the system through coupled energetic and transport contributions: while activation barriers determine intrinsic chemical reactivity, radical generation frequency, and interfacial mass transfer ultimately dictate the effective propagation and grafting rates. The integrated Arrhenius treatment of both initiator decomposition and reaction kinetics thus provides a coherent mechanistic basis for the temperature dependence summarized in Table 2.

### 3.7. Kinetic Modeling and Experimental Validation of Overall Polymerization and Grafting Conversions

**Figure 5:**
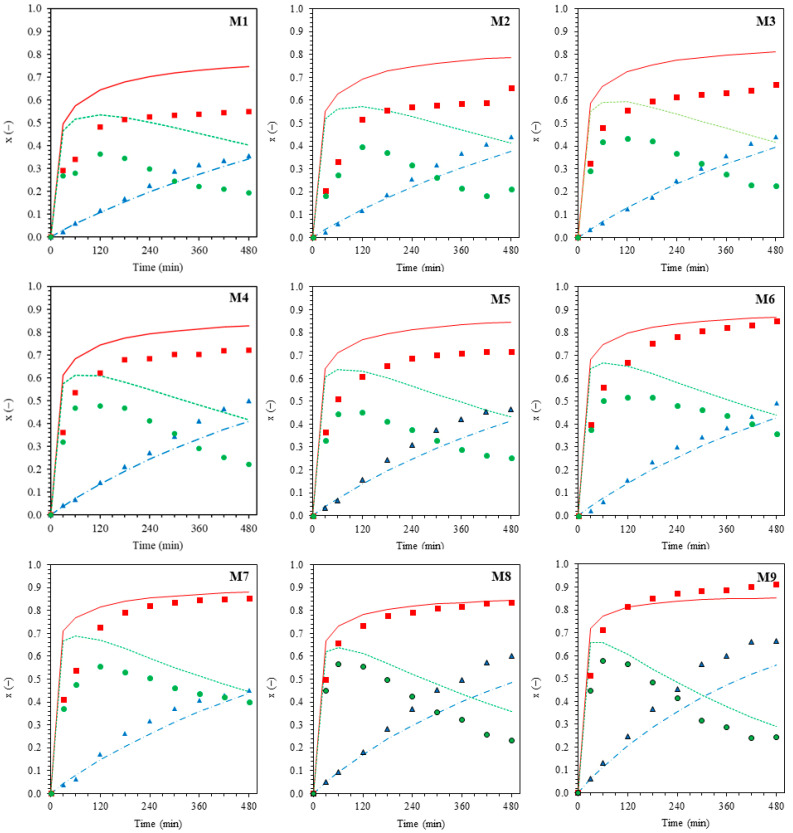
Time evolutions of *x_p_*, *x_g_*, and *x_f_* for the MMA-g-NR system under various conditions: (M1–M4) effect of initiator concentration ([CHPO] = 0.0051, 0.0102, 0.0154, and 0.0205 mol/L, respectively); (M3, M5, M6, M7) effect of monomer concentration ([MMA] = 0.39, 0.56, 0.71, and 0.83 mol/L, respectively); and (M3, M8, M9) effect of reaction temperature (T = 40, 50, and 60 °C, respectively); (see Table 1 for detailed conditions). Symbols represent experimental data (■: *x_p_*; ▲: *x_g_*; ⏺: *x_f_*), and solid lines denote model predictions (see Table 2). Red symbols and lines represent *x_p_*, blue symbols and lines represent *x_g_*, and green symbols and lines represent *x_f_*.

**Figure 6:**
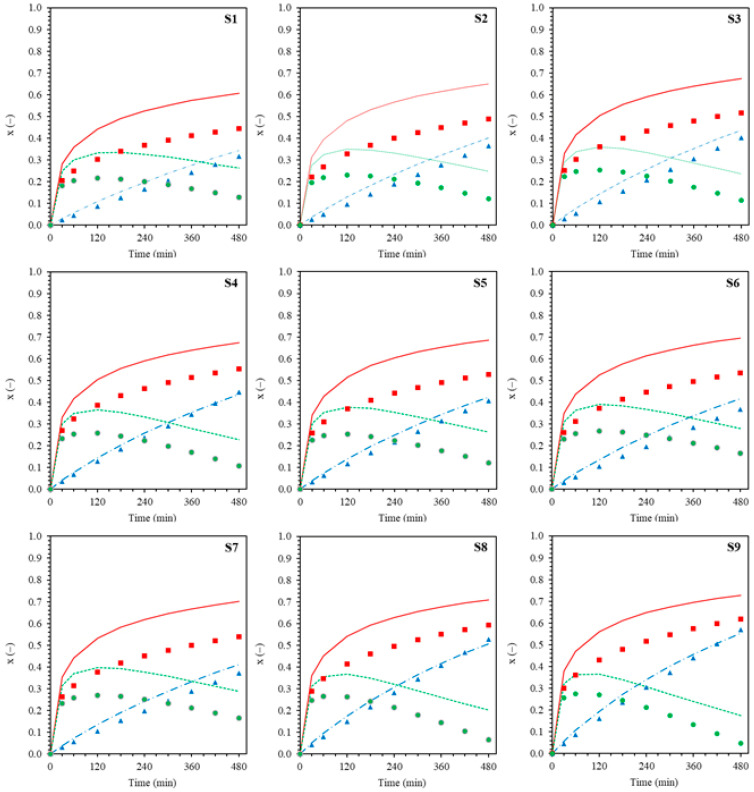
Time evolutions of *x_p_*, *x_g_*, and *x_f_* for the ST-g-NR system under various conditions: (S1–S4) effect of initiator concentration ([CHPO] = 0.0051, 0.0102, 0.0154, and 0.0205 mol/L, respectively); (S3, S5, S6, S7) effect of monomer concentration ([Styrene] = 0.39, 0.56, 0.71, and 0.83 mol/L, respectively); and (S3, S8, S9) effect of reaction temperature (T = 40, 50, and 60 °C, respectively); (see Table 1 for detailed conditions). symbols indicate experimental data (■: *x_p_*; ▲: *x_g_*; ⏺: *x_f_*), and solid lines denote model predictions (Table 2). Red symbols and lines represent *x_p_*, blue symbols and lines represent *x_g_*, and green symbols and lines represent *x_f_*.

The complete kinetic expressions for overall polymerization and grafting, including the temperature-dependent effective initiator term, are summarized in Table 2. These rate expressions define the time-dependent functions R_p_(t) and R_g_(t), which were formulated as ordinary differential equations in the general form d*x*/dt = R(t). Because the conversion time profiles exhibit nonlinear power-law behavior, the rate expressions explicitly include time dependence to represent the evolving radical population and monomer availability during the graft copolymerization process. The resulting equations were solved using the fourth-order Runge–Kutta (RK4) method, and the calculated conversions were sampled at the experimental reaction times. As shown in Figure 5 and Figure 6, the model predictions agree well with the experimental time evolutions of the overall polymer conversion (*x_p_*), graft copolymer conversion (*x_g_*), and free homopolymer fraction (*x_f_*) for the MMA-g-NR (M1–M9) and styrene-g-NR (S1–S9) systems.

For the MMA-g-NR system (Figure 5), increasing CHPO or MMA concentration at 313 K markedly accelerates *x_p_*, whereas *x_g_* increases more gradually due to the interfacial nature of the grafting process. Consequently, the separation between *x_p_* and *x_g_* becomes more pronounced at early reaction times, resulting in an increase in the free polymer fraction (*x_f_* = *x_p_* − *x_g_*). At longer reaction times, *x_f_* reaches a maximum and subsequently decreases as *x_p_* approaches a plateau while *x_g_* continues to increase. Increasing the temperature from 313 to 333 K further amplifies this behavior by preferentially accelerating *x_p_*. In contrast, the styrene-g-NR system (Figure 6) exhibits a more balanced evolution of *x_p_* and *x_g_* under comparable variations in initiator and monomer concentrations. This behavior reflects the preferential partitioning of nonpolar styrene into the rubber phase, which enhances interfacial grafting. As a result, increasing the temperature accelerates both conversions, with a less pronounced buildup of the free homopolymer fraction than in the MMA system.

**Figure 7:**
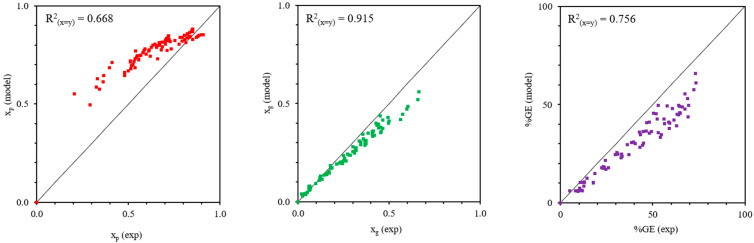
Experimental (symbols) and modeled (solid lines) profiles of *x_p_*, *x_g_*, and %GE for the MMA-g-NR system.

**Figure 8:**
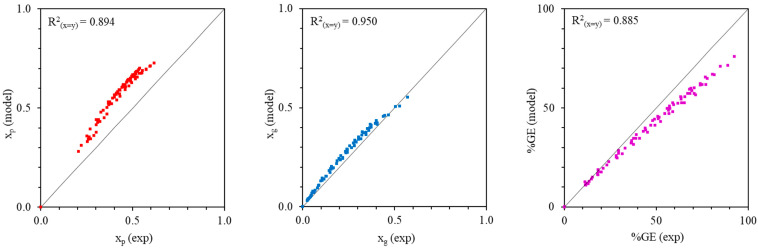
Experimental (symbols) and modeled (solid lines) profiles of *x_p_*, *x_g_*, and %GE for the styrene-g-NR system.

Figure 7 and Figure 8 show the statistical validation of the model predictions against the experimental data. For the MMA-g-NR system (Figure 7), the coefficients of determination (R^2^) between experimental data and model predictions are 0.668 for *x_p_*, 0.915 for *x_g_*, and 0.756 for %GE. This reduced correlation for *x_p_* in the MMA system can be attributed to its higher polarity and good water solubility, which promote homopolymerization in the aqueous phase, forming low-molar-mass, short-chain polymers that are difficult to precipitate and recover during gravimetric analysis, thereby contributing to increased deviations between experimental and predicted values. The lower R^2^ value for *x_p_* suggests larger deviations at higher overall conversions, which may arise from unaccounted effects such as viscosity buildup, monomer depletion, and diffusion limitations at advanced stages of the reaction. In contrast, the higher R^2^ value for *x_g_* indicates that the proposed model reasonably captures interfacial grafting kinetics, while the moderate agreement in %GE reflects the cumulative propagation of uncertainties associated with both *x_p_* and *x_g_*. For the styrene-g-NR system (Figure 8), higher R^2^ values of 0.894 for *x_p_*, 0.950 for *x_g_*, and 0.885 for %GE were obtained, indicating stronger agreement between experimental observations and model predictions. This improved agreement is attributed to the preferential partitioning of nonpolar styrene into the rubber phase, which promotes interfacial grafting and better satisfies the assumptions incorporated in the kinetic framework. Overall, these results demonstrate that the proposed model more effectively describes graft copolymerization systems involving rubber-phase–localized monomers. The high R^2^ values reflect strong consistency between the mechanistically derived kinetic model and the time-resolved experimental conversion profiles over the entire reaction period.

## 4. Conclusions

This study provides a mechanistic and kinetic interpretation of vinyl monomer grafting onto natural rubber latex under CHPO/TEPA redox initiation, demonstrating that graft copolymer formation in dispersed rubber systems is inherently governed by interfacial radical generation, monomer diffusion, and phase partitioning effects. The nonlinear power-law behavior is evidenced by fractional time exponents: for MMA, n_p_ = 0.2313 and n_g_ = 0.9511, while for styrene, n_p_ = 0.2642 and n_g_ = 0.9223. The markedly lower propagation exponent relative to grafting reflects the early onset of diffusion and termination constraints in the bulk phase, whereas graft formation proceeds cumulatively under interfacial control. The contrasting behaviors of MMA and styrene further highlight the decisive role of monomer polarity and rubber-phase affinity. Although styrene exhibits lower activation energies for both propagation and grafting (E_p_ = 4.64 and E_g_ = 10.76 kJ mol^−1^) compared with MMA (E_p_ = 10.94 and E_g_ = 20.14 kJ mol^−1^), its substantially smaller pre-exponential factors (A_p_ = 1.30 and A_g_ = 0.26) relative to MMA (A_p_ = 37.43 and A_g_ = 7.98) indicate frequency-limited radical encounters within the rubber phase. These quantitative differences demonstrate that graft efficiency is not governed solely by apparent activation barriers but also by interfacial accessibility and the frequency of radical collisions within the heterogeneous latex structure. Collectively, the kinetic exponents and Arrhenius parameters establish that multiphase NR systems are controlled by coupled energetic and transport phenomena. The derived rate relationships therefore provide a unified quantitative framework for understanding propagation–grafting competition in redox-initiated heterogeneous graft polymerization systems.

## Figures and Tables

**Table 1:** Recipes and polymerization conditions.

Run No.	Monomer Type	Run No.	Monomer Type	Initiator (mol/L)	T (°C)	Monomer (mol/L)	Time (min)
M1	MMA	S1	Styrene	0.0051	40	0.39	0–480
M2	MMA	S2	Styrene	0.0102	40	0.39	0–480
M3	MMA	S3	Styrene	0.0154	40	0.39	0–480
M4	MMA	S4	Styrene	0.0205	40	0.39	0–480
M5	MMA	S5	Styrene	0.0154	40	0.56	0–480
M6	MMA	S6	Styrene	0.0154	40	0.71	0–480
M7	MMA	S7	Styrene	0.0154	40	0.83	0–480
M8	MMA	S8	Styrene	0.0205	50	0.39	0–480
M9	MMA	S9	Styrene	0.0205	60	0.39	0–480

**Table 2:** Complete kinetic expressions for the overall polymerization and grafting of MMA and styrene.

System	Rate Expression
The effective initiator concentration (MMA system)	${\left[CHPO\right]}_{eff}=k_{d}\left(T\right){\left[CHPO\right]}_{0}=(3.48\times 10^{6}exp(-\frac{52,990}{RT}){\left[CHPO\right]}_{0}$
MMA—Overall polymerization (R_p_,_MMA_)	$R_{p,MMA}=37.43\;t^{-0.7687}\;([CHPO{]}_{eff}{)}^{0.2008}\;[MMA{]}_{0}^{1.353}\;exp(-\frac{10,936}{RT})$
MMA—Grafting (R_g,MMA_)	$R_{g,MMA}=7.98\;t^{-0.0489}\;[CHPO{]}_{0}^{0.1717}\;[MMA{]}_{0}^{1.1717}\;\exp(-\frac{20,139}{RT})$
The effective initiator concentration (Styrene system)	${\left[CHPO\right]}_{eff}=k_{d}\left(T\right){\left[CHPO\right]}_{0}=(6.70\times 10^{11}exp(-\frac{86,180}{RT}){\left[CHPO\right]}_{0}$
Styrene—Overall polymerization (R_p,ST_)	$R_{p,ST}=1.30\;t^{-0.7358}\;([CHPO{]}_{eff}{)}^{0.1637}\;[ST{]}_{0}^{1.0596}\;exp(-\frac{4646}{RT})$
Styrene—Grafting (R_g,ST_)	$R_{g,ST}=0.26\;t^{-0.0777}\;[CHPO{]}_{0}^{0.2681}\;[ST{]}_{0}^{0.953}\;exp(-\frac{10,762}{RT})$

Note: R_p_ and R_g_ are expressed in mol L^−1^ min^−1^, t in min, and the concentrations of monomer and CHPO in mol L^−1^. The units of the pre-exponential factors are: 37.43 (mol^−0.554^L^0.554^min^−0.2313^), 7.98 (mol^−0.3515^L^0.3515^min^−0.9511^), 1.30 (mol^−0.2239^L^0.2239^min^−0.2642^), and 0.26 (mol^−0.2211^L^0.554^min^−0.9223^).

## Data Availability

The data presented in this study are available on request from the corresponding author.

## Abbreviations

The following abbreviations are used in this manuscript:

AA	Acrylic acid
A_g_	Pre-exponential factor for grafting
AN	Acrylonitrile
A_p_	Pre-exponential factor for overall polymerization
a_g_	Reaction order with respect to initiator (graft copolymerization)
a_p_	Reaction order with respect to initiator (overall polymerization)
BPO	Benzoyl peroxide
b_g_	Reaction order with respect to monomer (graft copolymerization)
b_p_	Reaction order with respect to monomer (overall polymerization)
CAN	Ceric ammonium nitrate
CHPO	Cumene hydroperoxide
DHPVC	Dehydrochlorinated Poly(vinyl chloride)
DI	Deionized
DRC	Dry rubber content
EMA	Ethyl methacrylate
FTIR	Fourier transform infrared spectroscopy
GE	Grafting efficiency
GMA	Glycidyl methacrylate
KOH	Potassium hydroxide
MA	Maleic anhydride
MMA	Methyl methacrylate
NR	Natural rubber
NRL	Natural rubber latex
n_g_	Time exponent for graft copolymerization
n_p_	Time exponent for overall polymerization
PLA	Polylactic Acid
PMMA	Poly (methyl methacrylate)
PS	Polystyrene
PST	Polystyrene (used in grafted form context)
R	Rubber
RAFT	Reversible Addition–Fragmentation chain Transfer
R_g_	Grafting rate
R_p_	Overall polymerization rate
SDS	Sodium lauryl sulfate
ST	Styrene
St	Starch
TEPA	Tetraethylenepentamine
TEM	Transmission electron microscopy
UATR	Universal Attenuated Total Reflectance
*x_f_*	Free homopolymer conversion
*x_g_*	Graft copolymer conversion
*x_p_*	Overall polymer conversion
